# Age-Dependent Gut Microbiome Dysbiosis in Autism Spectrum Disorder and the Role of Key Bacterial Ratios

**DOI:** 10.3390/nu17111775

**Published:** 2025-05-23

**Authors:** Tanya Kadiyska, Dimitar Vassilev, Ivan Tourtourikov, Stanislava Ciurinskiene, Dilyana Madzharova, Maria Savcheva, Nikolay Stoynev, Rene Mileva-Popova, Radka Tafradjiiska-Hadjiolova, Vanyo Mitev

**Affiliations:** 1 Department of Physiology and Pathophysiology, Medical University, 1431 Sofia, Bulgaria; nstoynev@medfac.mu-sofia.bg (N.S.); rmpopova@medfac.mu-sofia.bg (R.M.-P.); rhadjiolova@medfac.mu-sofia.bg (R.T.-H.); 2 Genetic Medico-Diagnostic Laboratory Genica and Genome Center Bulgaria, 1612 Sofia, Bulgaria; mit.vassilev2826@gmail.com (D.V.); itourtourikov@gmail.com (I.T.); dmadzharova2205@gmail.com (D.M.); 3 Faculty of Biology, Sofia University “St. Kliment Ohridski”, 1504 Sofia, Bulgaria; 4 Department of Medical Chemistry and Biochemistry, Medical University, 1431 Sofia, Bulgaria; vmitev@mu-sofia.bg; 5 Vsyaka Duma Society, 1000 Sofia, Bulgaria; stanislava.ciurinskiene@abv.bg; 6 Department of Clinical Immunology, UMHATEM “N.I.Pirogov”, 1606 Sofia, Bulgaria; office@biomedical.bg

**Keywords:** autism spectrum disorder, dysbiosis, gut–brain axis, microbiome, biomarkers

## Abstract

**Background/Objectives**: Autism spectrum disorder (ASD) has a wide-ranging impact on individuals’ quality of life and development, and there is a critical need for greater awareness, early intervention, and comprehensive support strategies to effectively address the unique needs of those affected by ASD. Recent studies highlight the gut microbiome’s potential role in modulating ASD symptoms via the gut–brain axis, but specific microbial biomarkers remain unclear. This study aims to investigate differences in gut microbiota composition between ASD patients and neurotypical controls in a novel approach, specifically assessing ratios of Firmicutes/Bacteroidetes (F/B), Actinobacteria/Proteobacteria (A/P), and *Prevotella/Bacteroides* (P/B) as potential biomarkers. **Methods**: We analyzed gut microbiome samples from 302 Bulgarian children and adolescents diagnosed with ASD (aged 2–19 years). Microbial ratios (F/B, A/P, and P/B) were calculated and compared against previously reported reference meta-analytic means from European neurotypical populations. The statistical significance of deviations was assessed using parametric (*t*-tests), non-parametric (Wilcoxon signed-rank tests), and proportion-based (binomial tests) methods. Effect sizes were quantified using Cohen’s d. Significant differences between ASD cases and neurotypical reference values were observed across several age groups. **Results**: Notably, children with ASD demonstrated significantly lower F/B and A/P ratios, with the youngest cohort (0–4 years) exhibiting the greatest differences. Deviations in the P/B ratio varied across age groups, with a significant elevation in the oldest group (≥10 years). Collectively, ASD cases consistently exhibited microbiota profiles indicative of dysbiosis. **Conclusions**: Our findings support gut microbiome dysbiosis as a potential biomarker for ASD, highlighting significantly altered bacterial ratios compared to neurotypical controls. These microbiome shifts could reflect early-life disruptions influencing neurodevelopment. Future studies should adopt longitudinal and mechanistic approaches to elucidate causal relationships and evaluate therapeutic microbiome modulation strategies.

## 1. Introduction

ASD encompasses a heterogeneous group of neurodevelopmental disorders characterized by delays in developmental milestones, deficits in social communication and interaction, presence of repetitive and restrictive behaviors, and challenges in peer relationships [1]. According to the US Centers for Disease Control (CDC), the global prevalence of ASD is about 1 in 100 children as of 2024, and for Bulgaria, 875 in every 100,000. The diverse etiological factors and clinical presentations associated with ASD complicate the diagnostic process and subsequent therapeutic interventions, posing significant challenges for affected individuals and their families. Recently, there have been many attempts to find an “ASD cause”, ranging from genetic predisposition to environmental exposure, but no single causative agent can be identified [2]. Increasing evidence suggests the role of the gut–brain axis in the pathophysiology of neuropsychiatric and neurological disorders. The relationship between gut dysbiosis, which is frequently observed in ASD patients, and the modulation of brain function and social behavior has been widely described [3]. Individuals with ASD often experience gastrointestinal disturbances, such as chronic diarrhea, constipation, and abdominal pain, which have been correlated with distinct alterations in gut microbiome composition [4]. Similarly, these children were five times more likely to develop feeding problems such as food selectivity and refusal, and showed a preference for carbohydrates and processed foods, which in turn further disrupts the normal bacterial composition [5]. In addition, gastrointestinal dysbiosis may lead to the synthesis of dangerous metabolites and neuroinflammation, which exacerbate behavioral and cognitive deficits [6].

The human gastrointestinal tract (GIT) harbors a vast number of bacterial cells, which outnumber the host’s cells by a factor of 10 [7]. The extensive diversity of microbes inhabiting the GIT and their mutualistic relationship with the human host are referred to as the gut microbiome, integral to nutrient metabolism, pharmacokinetics, host defense, systemic immunity, and neurodevelopment [8,9]. Deviations from eubiosis have been linked to gastrointestinal disease, metabolic disorders (obesity, diabetes), and neuropsychiatric conditions including anxiety, schizophrenia, anorexia nervosa, and ASD [7,8,9]. Its composition is shaped by delivery mode, diet, lifestyle, and host genetics [10]. In children with ASD, multiple studies document dysbiosis, heightened intestinal permeability, and a correlation between gastrointestinal symptom severity and behavioral severity [11,12].

These observations fit within the gut–brain axis (GBA), the bidirectional network connecting the central and enteric nervous systems through neural, endocrine, immune, and humoral pathways [6,12]. This communication is facilitated by endocrine, humoral, neural, and immune connections between the CNS and the GIT [6]. Microbial metabolites—short-chain fatty acids (SCFAs; acetate, propionate, butyrate), aromatic compounds, free amino acids, and neurotransmitters such as γ-aminobutyric acid (GABA)—are key signaling molecules [6,13]. These metabolites communicate with the brain via the vagus nerve and endocrine system [6]. One proposed mechanism by which the microbiota interact with the GBA is through “modulation of afferent sensory nerves, enhancing their excitability by inhibiting calcium-dependent potassium channels opening, thus modulating gut motility” [14]. SCFAs, which are the primary metabolites produced by the gut microbiota, play a critical role in facilitating communication between the gut and the central nervous system, as they possess the ability to traverse the blood–brain barrier (BBB) [15]. SCFAs cross the blood–brain barrier, modulate sympathetic tone, stimulate mucosal serotonin release, and influence cognition and memory; excessive propionate has been implicated in oxidative stress and neurodegeneration [14,16]. Children with ASD show elevated fecal levels of several SCFAs compared with neurotypical controls [17]. GABA is the main inhibitory neurotransmitter, with a calming and anxiolytic effect on the CNS [6,13]. Many gut taxa (e.g., *Bifidobacterium*, *Lactobacillus*, and *Bacteroides*) carry GAD genes, enabling GABA synthesis [18,19]. When the microbial balance is disrupted, altered metabolite profiles can provoke immune activation, endocrine change, and vagal signaling, thereby affecting mood, cognition, and behavior [20]. Microbial colonization of the gut begins at birth [21]. As the neonate traverses the birth canal, it is first exposed to the microbial flora residing in the maternal vaginal microbiome. Subsequently, infants delivered through cesarean section show reduced microbial development after the first month, when compared to those delivered naturally. Further alterations in microbial composition occur as the infant transitions from breastfeeding to formula feeding and eventually to the introduction of solid foods. Despite these factors influencing variations during the first year of life, the overall composition in subsequent years is relatively stable at the phylum level. Environmental and genetic determinants significantly contribute to the establishment of the bacterial composition in children during the critical first 1000 days postnatally, extending into adulthood [22]. Between 75 and 90% of microbial species belong to Firmicutes, Bacteroidetes, Actinobacteria, Fusobacteria, Verrucomicrobia, and Proteobacteria [23]. In the case of ASD, this microbial balance is often disrupted; according to a study by Fattorusso et al., “[ASD is characterized by] an increase in microflora and decrease in microbial diversity” [24]. This disrupted phylum-level balance in ASD is further characterized by significant alterations in the abundance of specific bacterial families and species, which will be examined in detail to elucidate their potential roles in the pathophysiology of the disorder.

Firmicutes, a gram-positive phylum of bacteria, account for more than 50% of microbial species inhabiting the gut. Well-known constituents of this phylum include *Staphylococcus*, *Enterococcus*, *Clostridium*, *Faecalibacterium*, as well as *Lactobacillus*, an important probiotic species. They participate, namely, in the fermentation of dietary fibers and the production of SCFAs, such as butyrate [23]. According to Sun et al., Firmicutes may play an important role in maintaining host homeostasis by mitigating gut permeability and inflammation via breaking down dietary fibers [25]. The relative levels of Firmicutes in the gut, especially compared to Bacteroidetes, can allude to the potential development of various metabolic diseases, such as obesity and type 2 diabetes [26]. Generally, an elevated or decreased F/B ratio indicates dysbiosis; its normal reference values are less than 1.5 [27]. Increased levels of Firmicutes and lower levels of Bacteroidetes have been associated with obesity and other metabolic disease states, whereas the opposite ratio is associated with irritable bowel syndrome (IBS), when compared to healthy controls [26,28].

Accounting for about 25% of the gut microbiome, Bacteroidetes are particularly important in carbohydrate fermentation. This gram-negative phylum includes *Bacteroides* and *Prevotella* [29]. The relative proportions of *Prevotella* and *Bacteroides (P/B)* are a recognized biomarker of dietary influence and metabolic homeostasis that has also been associated with gastrointestinal inflammation in ASD; this ratio determines the enterotype of the person [30]. Elevated *Bacteroides*, or enterotype 1, are generally associated with “western” diets that are high in fat and protein, whereas *Prevotella* (enterotype 2) are more abundant in Asian populations, where diets are considered more “plant-rich”. *Prevotella* plays a crucial role in fermenting complex plant-derived carbohydrates to produce SCFAs that support gut barrier integrity and modulate immune responses [31]. *Prevotella* species are more abundant in individuals consuming high-fiber, plant-rich diets, and reduced levels have been reported in some ASD cohorts, which may lead to lower SCFA production and compromised gut permeability [31,32]. *Bacteroides* may serve to protect from pathogens, as well as deliver nutrients to other microbes in the gut [29]. *Bacteroides* are essential for degrading complex carbohydrates and proteins, thereby facilitating nutrient absorption and energy metabolism. These gram-negative anaerobes are typically associated with Western diets high in fats and proteins, and several studies have found an increased abundance of *Bacteroides* in children with ASD compared to neurotypical controls. Elevated *Bacteroides* levels can lead to higher production of propionic acid—a short-chain fatty acid that, at high concentrations, may disrupt gut homeostasis, trigger peripheral inflammation, and affect neurotransmitter production by crossing the blood–brain barrier [33]. *Bacteroides* are also major producers of propionic acid, which in high concentrations “alters gut homeostasis, triggers peripheral inflammation” and can reach the brain via the BBB, where it affects serotonin and dopamine production [34].

The phylum Actinobacteria represents a relative minority in the gut microbiome, but their role is of no less significance. Studies show their involvement in the modulation of gut permeability, stimulation of the immune system, as well as metabolism through their production of secondary metabolites [35]. *Bifidobacteria* are an important genus within this phylum, benefiting the host through their production of GABA and helping to digest fibers and prevent infections [36]. This bacterial genus has been associated with numerous health benefits, including improved lactose digestion, enhanced immune responses, and a reduced risk of allergic diseases [37]. Patients with ASD tend to show generally lower levels of Actinobacteria when compared to healthy controls [11]. The decreased levels of *Bifidobacteria* in children with ASD correlate proportionally to observed decreased amounts of GABA in the brains of the same children [38]. *Bifidobacteria* also have a role in bile production and tryptophan synthesis, the latter of which is a precursor in the synthesis of serotonin [39]. A decrease in *Bifidobacteria* may lead to a decrease in serotonin in the brain, which has also been associated with autistic behavior [38].

Proteobacteria, a phylum of gram-negative bacteria, comprises many known human pathogens such as *Haemophilus* spp., *Klebsiella*, *Enterobacter*, *Pseudomonas* spp., and many more. They are present in the healthy human gut in small proportions, relative to other bacteria [40]. Several studies identify elevated levels of Proteobacteria in the GIT as a hallmark of diseases, mostly of an inflammatory phenotype. Their presence creates dysbiosis in the gut and produces inflammation, which further deepens symptoms such as irritability, anxiety, and social withdrawal [41]. In children with ASD, there is an abundance of Proteobacteria in the gut, which contributes to host inflammation [27]. Interestingly, particularly elevated levels of this phylum seemed to correlate with the severity of ASD; those showing ASD with mental regression had higher values of Proteobacteria in feces [42]. As they are opportunistic pathogens, elevations in their levels lead to reductions in beneficial bacteria, such as Actinobacteria [40]. The ratio of Actinobacteria to Proteobacteria also serves as a biomarker for gut health and homeostasis [35]. In children with ASD, this ratio is significantly reduced [43].

Altered microbiome ratios have been linked to increased intestinal permeability and immune dysregulation, mechanisms that contribute to ASD pathophysiology via the gut–brain axis [44]. Microbiome ratios, such as the F/B, have been examined in connection with ASD, as well as overall changes in bacterial abundance of beneficial Actinobacteria and pathogenic Proteobacteria, but to our knowledge, this is the first study to examine the combined utility of the following three specific ratios as a potential biomarker [45]. These three ratios appear with conflicting results throughout the literature; furthermore, most have focused solely on the F/B or studied these ratios separately and it is necessary to clarify their impact and significance in patients with ASD as a comprehensive microbiome profile, as well as their potential use as therapeutic biomarkers or targets, either individually or in combination [46]. Keeping this in mind, along with the need to enhance awareness, implement early intervention, and develop comprehensive, individualized support strategies for those with ASD, the aim of this study is to search for a correlation between ASD and changes in the F/B, A/P, and P/B ratios and the possibility for these ratios to serve as a multidimensional biomarker for ASD. For this goal, we examined the microbiomes of 302 patients with a confirmed ASD diagnosis, aged 2–19, and compared them to neurotypical children of the same age, as reference values.

## 2. Materials and Methods

A total of 302 Bulgarian children (230 males and 72 females), ages 2–19 years, were included in this study. All participants were diagnosed with ASD based on the Diagnostic and Statistical Manual of Mental Disorders, Fifth Edition (DSM-5; American Psychiatric Association, 2013), followed by a developmental assessment performed by a psychologist administering the diagnostic pipeline implemented in the Developmental Profile-3 (DP-3) test (© 2007 by Western Psychological Services 12031 Wilshire Blvd., Los Angeles, CA 90025-1251, USA). DP-3 is a standardized diagnostic assessment tool, designed for use to assess children from birth through to 12 years, 11 months. The algorithm measures development across five scales: physical, adaptive behavior, social-emotional, cognitive, and communication. To ensure diagnostic consistency, assessments also included developmental history, behavioral observations, and standardized diagnostic instruments where applicable.

### Sample Collection and Processing

To evaluate the gut microbiome, stool samples were collected from all study participants in sterile containers provided by the laboratory. All participants or their parents/legal guardians completed informed consent forms, which included a clause regarding data protection in compliance with the General Data Protection Regulation (GDPR). Participants were required not to take antibiotics, probiotics, or prebiotics for at least 4 weeks before sample collection. After collection, the stool samples were immediately processed. DNA was extracted using the MutaCLEAN^®^ Universal RNA/DNA kit (Immundiagnostik AG, Bensheim, Germany) according to the manufacturer’s instructions.

After measuring the concentration of the extracted DNA, quantitative detection of bacterial DNA was performed using three real-time PCR kits from Immundiagnostik AG (Germany): MutaPLEX^®^ AKM/FAEP, MutaPLEX^®^ EU/BAC/BIF, and MutaPLEX^®^ PRE/RU/LA. These kits allow for the detection and quantification of key gut microbiota taxa and species, including *Akkermansia muciniphila* and *Faecalibacterium prausnitzii* (AKM/FAEP), *Bacteroides* spp., *Bifidobacterium* spp., and the *Eubacterium rectale* group (EU/BAC/BIF), as well as *Prevotella* spp. and *Ruminococcus* spp. from the families Ruminococcaceae and Lachnospiraceae (PRE/RU/LA). The reaction mixture for real-time PCR was prepared according to the manufacturer’s instructions, in a final volume of 20 µL, consisting of 16 µL master mix with 4 µL of extracted DNA. The prepared mixture was aliquoted into a 96-well PCR plate and loaded into the Real-Time PCR system (Rotor-Gene Q 5plex HRM, Qiagen, Hilden, Germany). Amplification was performed with the following thermal cycling conditions: initial denaturation at 95 °C for 5 min, followed by 40 cycles consisting of denaturation at 95 °C for 30 s and annealing/extension at 60 °C for 30 s. Detection channels were assigned according to target-specific probes and internal controls, including FAM, Cy5, ROX, and VIC/HEX. Standard curves generated using DNA standards ranging from 10^2^ to 10^6^ copies per reaction were used for quantification. A correction factor was applied to convert detected copy numbers to bacterial load per gram of stool. To assess microbial community composition, absolute read counts per taxon were normalized to relative abundances by dividing each taxon’s count by the total number of reads in the corresponding sample. The resulting proportions were multiplied by 100 to express values as percentages. Mean relative abundances were then calculated across all samples for each taxon to characterize average microbial profiles. Each run included a no-template control, a negative control, and a positive control to ensure assay validity. All DNA extractions and 16S-rRNA PCR amplifications were performed with the same commercial kits and the same lot numbers throughout the study period.

Following initial quality checks, individuals presenting extreme bacterial ratio values (>20) were excluded. To align with previously published meta-analytic findings by Deering et al., the cohort was partitioned into five age categories: <4 years, 4–<6 years, 6–<8 years, 8–<10 years, and ≥10 years. In addition, all 302 participants were collectively compared to findings from European population studies using the region-specific mean reported in the same meta-analysis. The three bacterial ratio metrics were calculated for each participant. For both ratios, we computed (i) the mean within each age bin, (ii) the standard deviation (SD), and (iii) the number of valid samples (Supplementary File S1). The published means from Deering et al. were used as fixed references, owing to the absence of variance estimates in that meta-analysis. Each bin underwent one-sample *t*-tests to detect significant departures from the corresponding reference mean. In parallel, Wilcoxon signed-rank tests accounted for skewed distributions, and binomial signed tests quantified whether more than half of the samples lay above or below the reference. Effect sizes were estimated via Cohen’s d, calculated by subtracting the meta-analytic mean from the bin’s observed mean and dividing by the bin’s SD. Confidence intervals (95%) around each bin’s mean were generated using the *t*-distribution. Proportions of samples above, below, or tied with the reference mean were determined for additional context on distributional shifts. Scatterplots were created to visualize each data point relative to the published mean, providing a graphical depiction of group-level deviations.

## 3. Results

### 3.1. Firmicutes/Bacteroidetes Ratios

**Figure 1:**
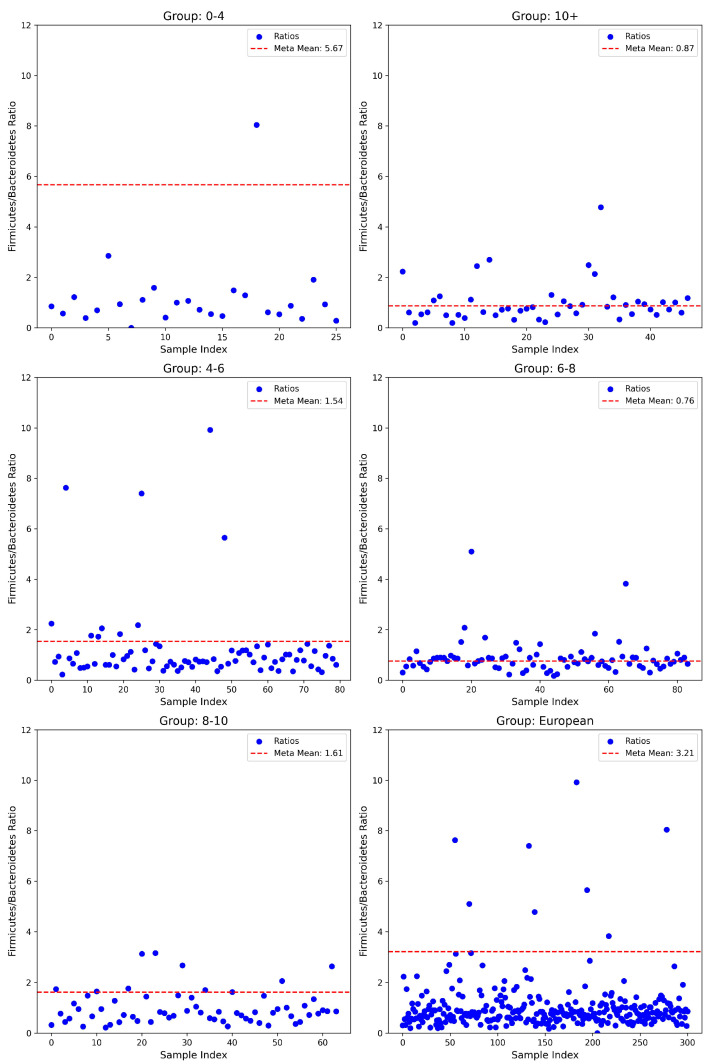
Firmicutes/Bacteroidetes ratio of ASD cases vs. meta-means among different age groups of the overall European population.

Evaluation of the F/B ratio demonstrated distinct age-related variations. In the youngest population (0–4 years, *n* = 26), a notably lower mean ratio (1.18 ± 1.52) was observed compared to the meta-mean of 5.67. This deviation was statistically significant (*t*-test, *p* = 4.72 × 10^−14^; Wilcoxon test, *p* = 5.96 × 10^−8^; Binomial test, *p* = 8.05 × 10^−7^), alongside a robust negative effect size (Cohen’s d = −2.96). In the subsequent age group of 4–6 years (*n* = 80), the mean ratio (1.22 ± 1.58) was slightly below the meta-mean (1.54), showing marginal statistical significance in parametric testing (*t*-test, *p* = 0.074), yet highly significant differences emerged with non-parametric methods (Wilcoxon test, *p* = 8.03 × 10^−9^; Binomial test, *p* = 3.16 × 10^−12^), albeit with a relatively smaller negative effect size (Cohen’s d = −0.20). Interestingly, the ratio appeared to equilibrate in children aged 6–8 years (0.87 ± 0.67; *n* = 84), closely matching the meta-mean (0.76) with no significant deviation detected (*t*-test, *p* = 0.123; Wilcoxon test, *p* = 0.855; Binomial test, *p* = 0.912; Cohen’s d = 0.17). Conversely, a significant reduction was again, observed in the 8–10 years group (0.99 ± 0.66; *n* = 64) compared to the meta-mean of 1.61, supported by highly significant statistical findings (*t*-test, *p* = 2.11 × 10^−10^; Wilcoxon test, *p* = 1.25 × 10^−7^; Binomial test, *p* = 2.00 × 10^−8^) and a robust negative effect size (Cohen’s d = −0.94). Finally, the comparison of the entire cohort to the available European dataset (*n* = 301) exhibited a considerably lower mean ratio (1.03 ± 1.09) relative to the significantly higher meta-mean (3.21, *t*-test, *p* = 1.17 × 10^−106^; Wilcoxon test, *p* = 4.45 × 10^−45^; Binomial test, *p* = 7.68 × 10^−76^) and a prominent negative effect size (Cohen’s d = −1.99) (see Figure 1).

### 3.2. Actinobacteria/Proteobacteria Ratios

**Figure 2:**
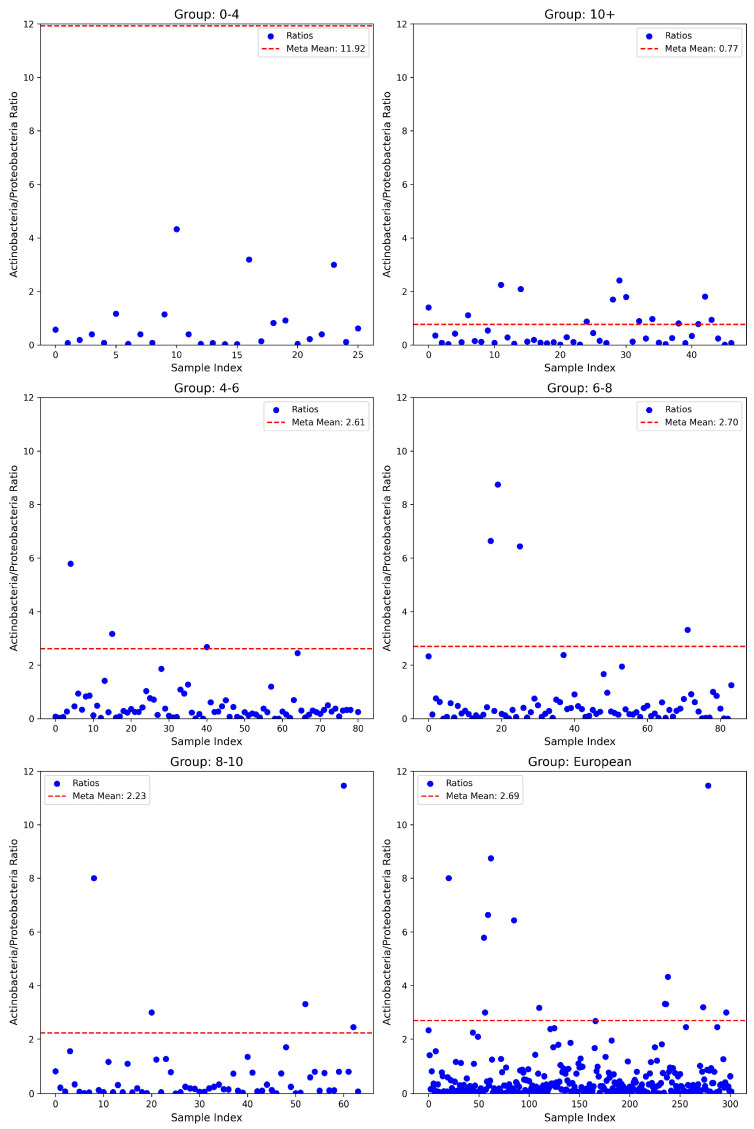
Actinobacteria/Proteobacteria ratio of ASD cases vs. meta-means among different age groups of the overall European population.

The analysis of the A/P ratio revealed significant and consistent deviations from the corresponding meta-mean across all age groups. In the youngest cohort (0–4 years), the observed mean ratio was notably lower (0.71 ± 1.10; *n* = 26) compared to the reported meta-mean of 11.92, representing a highly significant difference validated by both parametric (*t*-test, *p* = 5.86 × 10^−27^) and non-parametric tests (Wilcoxon test, *p* = 2.98 × 10^−8^; Binomial test, *p* = 2.98 × 10^−8^). The associated effect size, quantified by Cohen’s d, was substantial at −10.16, underscoring a biologically meaningful deviation. Similarly, the 4–6 years age group exhibited a lower ratio (0.67 ± 1.61; *n* = 81) compared to the meta-mean of 2.61, again supported by statistical significance across multiple tests (*t*-test, *p* = 2.49 × 10^−17^; Wilcoxon test, *p* = 1.90 × 10^−12^; Binomial test, *p* = 1.45 × 10^−18^) and a large negative effect size (Cohen’s d = −1.20). The trend of significantly lower ratios was consistently observed in the 6–8 years cohort (0.69 ± 1.41; *n* = 84; meta-mean = 2.70), evidenced by stringent significance levels (*t*-test, *p* = 6.90 × 10^−22^; Wilcoxon test, *p* = 8.07 × 10^−12^; Binomial test, *p* = 2.10 × 10^−19^) and a substantial effect size (Cohen’s d = −1.43). Likewise, children aged 8–10 years demonstrated a reduced mean ratio (0.78 ± 1.79; *n* = 64) compared to their respective meta-mean (2.23), with significant differences across statistical methods (*t*-test, *p* = 1.55 × 10^−8^; Wilcoxon test, *p* = 1.78 × 10^−9^; Binomial test, *p* = 9.00 × 10^−13^) and a moderate effect size (Cohen’s d = −0.81). Importantly, the European cohort (*n* = 302) similarly presented a reduced mean ratio (0.68 ± 1.44) against the meta-mean of 2.69, demonstrating profound statistical significance (*t*-test, *p* = 1.46 × 10^−72^; Wilcoxon test, *p* = 3.00 × 10^−42^; Binomial test, *p* = 1.14 × 10^−67^) and a robust effect size (Cohen’s d = −1.39) (see Figure 2).

### 3.3. Prevotella/Bacteroides Ratios

**Figure 3:**
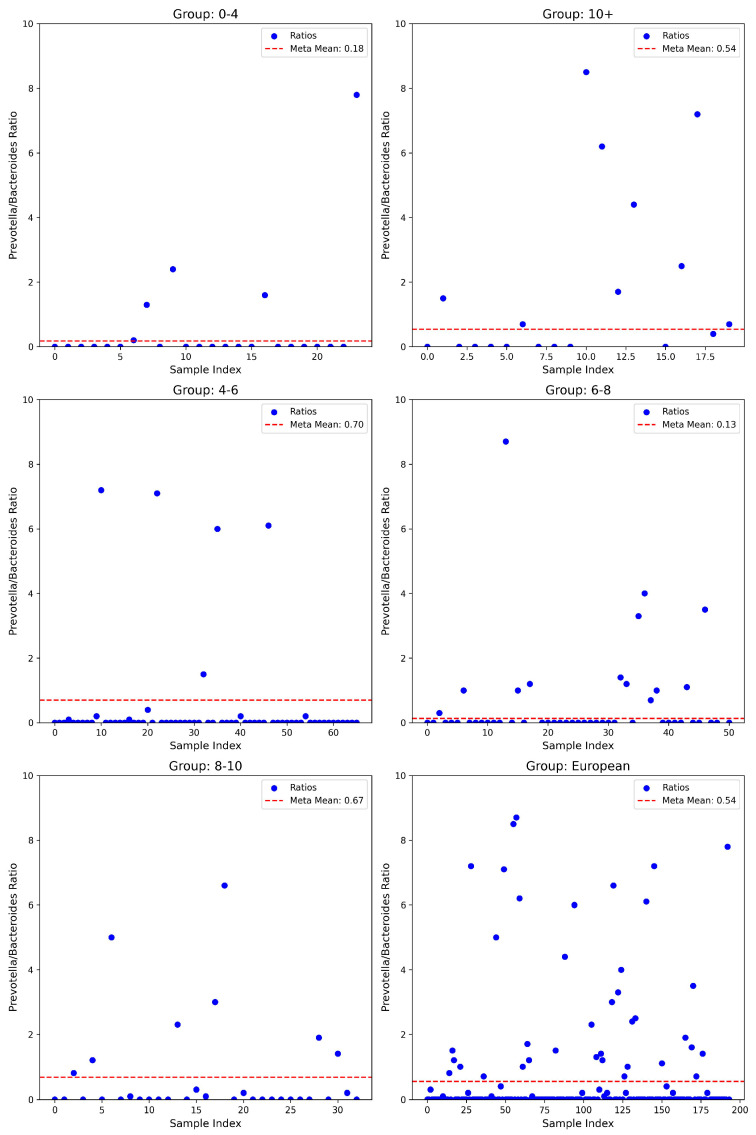
*Prevotella/Bacteroides* ratio of ASD cases vs. meta-means among different age groups of the overall European population.

The analysis of the P/B ratio uncovered considerable variations. Within the youngest age group (0–4 years, *n* = 24), the mean ratio (0.55 ± 1.66) exceeded the meta-mean of 0.18, although statistical tests yielded non-significant parametric results (*t*-test, *p* = 0.281), while non-parametric binomial testing indicated significance (*p* = 0.007), with a modest positive effect size (Cohen’s d = 0.23). Notably, the oldest cohort (≥10 years, *n* = 20) exhibited a pronounced elevation in ratio (2.44 ± 3.98) versus the meta-mean of 0.54, confirmed by significant parametric testing (*t*-test, *p* = 0.046), though non-parametric assessments indicated limited statistical significance (Wilcoxon test, *p* = 0.245; Binomial test, *p* = 1.00), accompanied by a moderate positive effect size (Cohen’s d = 0.48). The intermediate age group of 4–6 years (*n* = 66) had a slightly reduced mean ratio (0.44 ± 1.59) compared to the meta-mean (0.70), with significance primarily observed through non-parametric testing (Wilcoxon test, *p* = 6.12 × 10^−8^; Binomial test, *p* = 2.63 × 10^−13^), despite non-significant parametric results (*t*-test, *p* = 0.191; Cohen’s d = −0.16). The 6–8 years group (*n* = 51) displayed a significantly higher ratio (1.20 ± 3.52) relative to the meta-mean of 0.13, statistically validated by parametric and binomial tests (*t*-test, *p* = 0.034; Binomial test, *p* = 0.005), yet non-significant Wilcoxon results (*p* = 0.977) with a moderate positive effect size (Cohen’s d = 0.31). In European populations (*n* = 194), the mean ratio was elevated (0.91 ± 2.59) compared to a meta-mean of 0.54, approaching significance in parametric testing (*t*-test, *p* = 0.051), but with strong non-parametric confirmation (Wilcoxon test, *p* = 7.54 × 10^−6^; Binomial test, *p* = 2.14 × 10^−16^) and small positive effect size (Cohen’s d = 0.14) (see Figure 3).

## 4. Discussion

Our results suggest that children with ASD present markedly different ratios of F/B and A/P compared to European neurotypical individuals. Both ratios were generally shifted downward, often by one or more standard deviations. Across the examined microbial ratios, consistent age-dependent differences emerged, reflecting dynamic developmental shifts in the gut microbiota throughout childhood. The youngest participants showed especially pronounced deviations, hinting at possible early-life disruptions in gut microbiota composition. Although treating the meta-analytic means as fixed values likely overstates the certainty of the observed differences, the combination of parametric (*t*-test), non-parametric (Wilcoxon), and proportion-based (binomial) approaches consistently revealed significant discrepancies. The lack of difference between cases and controls for the F/B ratio in the 6–8 years old range is most likely due to the small sample size of the controls for this particular age group. Additionally, the reduction of beneficial bacteria such as Firmicutes, Actinobacteria, and Bacteroidetes allows opportunistic pathogens, such as those in Proteobacteria, to flourish. Concerning the amount of Proteobacteria present in ASD cases, an increase is observed compared to neurotypical controls in all five age groups. This confirms the claim that children with ASD show an increase in pathogenic bacteria, whose metabolites, in turn, may contribute to behavioral symptoms. The lack of difference between cases and controls for the F/B ratio in the 6–8 years old range is most likely due to the small sample size of the controls for this particular age group. Additionally, the reduction of beneficial bacteria such as Firmicutes, Actinobacteria, and Bacteroidetes allows opportunistic pathogens, such as those in Proteobacteria, to flourish. Concerning the amount of proteobacteria observed, an increase is observed in cases compared to controls, in all five age groups. This confirms the claim that children with ASD show an increase in pathogenic bacteria, whose metabolites, in turn, may contribute to symptoms.

Regarding the gut microbiomes of patients with ASD, there are conflicting results discussed in the literature regarding changes in this ratio; several studies observe an elevated F/B ratio, while others report the opposite [27]. According to Ronan et al., children with ASD have elevated Bacteroidetes and lower levels of Firmicutes, whereas Strati et al. found the opposite, with an increase in Firmicutes and a decrease in Bacteroidetes [47,48]. Similarly, Wong et al., which examined an Asian cohort with ASD, report a significantly higher F/B ratio when compared to neurotypical individuals [49]. These differences may be partly due to various nutritional habits, age, food availability, and geographical distribution of the studied cohorts. Finegold et al. report elevated levels of Firmicutes in the ASD cohort and decreased Bacteroidetes, but it is important to note their cohort is North American, with a significantly different diet than those in Bulgaria [50]. These contradictory results may also be due to screening differences and exclusions of certain microbial families in various studies; for example, Młynarska et al. reported both elevated Firmicutes and Bacteroidetes in ASD patients, but the focus was on specific species such as *Clostridia* and *Lactobacillus*, rather than the phylum Firmicutes as a whole [13,51,52]. In addition, confounding results may arise due to differences in the units of measurement, namely, whether bacterial abundance is reported as a mean relative abundance or as an absolute count. In our study, we rely on relative abundance, which expresses the proportion of each taxon as a percentage of the total microbial community. However, this approach introduces an important limitation: an increase in the relative abundance of one taxon can artificially imply a decrease in another, even if the latter’s absolute concentration has not changed. This compositional nature of relative abundance data means that shifts in the microbial profile must be interpreted with caution, as changes in one group can create the illusion of change in others simply due to the percentage-based representation [53].

Unlike the consistently reduced F/B and A/P ratios indicative of dysbiosis, the P/B ratio exhibited heterogeneous, age-dependent patterns, suggesting it may reflect more nuanced microbial shifts influenced by developmental, dietary, or environmental factors rather than a uniform signature of ASD-related dysbiosis. As previously mentioned, this ratio is used to establish enterotype, giving insight into the feeding habits of the individual. The non-standardized dietary difference among our cases may influence the observed patterns. Our results confirm what has been found in similar studies; an Israeli study also found an increase in *Bacteroides* in an autism cohort compared to neurotypical controls [54]. Our findings show that ASD cases present with a lowered P/B ratio in comparison to reported results for neurotypical individuals.

The greatest differences in cases compared to controls for both ratios were observed in the 0–4 age group. The microbiome during the first four years of life is greatly impacted by a wide variety of factors, which start with the mother—gestational diet, overall health during pregnancy, stress, perinatal environment, antibiotic, probiotic, and/or medication intake, as well as mode of delivery [55]. These factors make it difficult to describe a “normal” healthy microbiome during this age group. Additionally, the mode of feeding and the overall environment surrounding the child during the first two years of life also play a major role in how these proportions will develop later on. Whether the child was fed entirely on breast milk or formula, the time of shifting to solid foods, and the variety of foods given, as well as the number of caregivers during this period, all have an impact on the microbial diversity [56]. According to Ronan et al., the gut microbiome during the breastfeeding period is dominated by *Bifidobacteria* and *Lactobacillus*, and the number of dietary fibers in the weaning period also affects the ratios [48]. On the other hand, infants fed solely formula show higher levels of pathogenic bacteria such as *Clostridium difficile* and *Enterobacteriaceae* [57].

Taken together, the gut microbiota of individuals with ASD in this cohort displays three specific features of dysbiosis. First, the A/P ratio is persistently and substantially reduced across all age strata, signifying depletion of beneficial Actinobacteria and concomitant expansion of potentially pathogenic Proteobacteria. Second, the F/B ratio is broadly depressed but approaches normative values during mid-childhood, suggesting a transient, age-limited re-equilibration of community structure. Third, the P/B ratio follows a biphasic, age-contingent trajectory that likely reflects diet- and maturation-driven shifts in ecological niche partitioning. The concurrent suppression of A/P and F/B ratios during the neuro-developmentally sensitive preschool years strengthens the proposition that early-life losses of short-chain fatty acid-producing and vitamin-biosynthetic taxa—together with relative enrichment of endotoxin-producing Proteobacteria—may contribute to the metabolic and immunological perturbations observed in ASD. Rigorous longitudinal cohorts incorporating detailed dietary profiling will be essential to delineate causal pathways and to determine whether the targeted restoration of these ratios can ameliorate neurobehavioral phenotypes.

This study has several limitations that should be considered when interpreting the findings. First, the cross-sectional design precludes establishing a causal relationship between microbiome alterations and ASD symptoms. Without longitudinal data, it remains unclear whether the observed dysbiosis is a cause or a consequence of the restricted eating behaviors and other lifestyle factors common in ASD. Additionally, there is the potential for multiple confounders. Variables such as age, gender, medication use, and comorbid conditions (including gastrointestinal disorders and food allergies) may have influenced both dietary intake and gut microbiota composition. The lack of detailed dietary records and the lack of dietary exclusion criteria for the cohorts and controls further limit our ability to disentangle the effects of restricted diets from intrinsic microbial alterations. This confounding factor limits our ability to discern whether these changes in microbial ratios are due to ASD alone. Considering the restrictive feeding behaviors commonly associated with children diagnosed with ASD, it remains challenging to ascertain whether the observed microbial dysbiosis directly contributes to neurodevelopmental impairments or whether it is secondary to dietary restrictions. Methodologically, variations in sample collection and storage could include subtle biases. The use of meta-analytic reference values for comparison with our ASD cohort may also contribute to bias, as these external controls might not fully match our study population in terms of demographic and environmental factors. Because the benchmark meta-means lack variance estimates, our one-sample statistics assume zero measurement error in the reference values, potentially inflating significance. Consequently, the statistical significance we report should be interpreted with caution until population-specific reference ranges and their confidence intervals become available or are empirically derived in future studies. Furthermore, this study relied on a single stool sample from each participant, which may not adequately capture the temporal variability of the gut microbiome. Moreover, it is important to emphasize that the relationship between gut microbiota and behavior is likely bidirectional. Not only may restricted diets shape the microbial community but altered microbiota composition could also affect appetite regulation and food preferences, potentially establishing a vicious cycle. Furthermore, our cross-sectional study design limits causal inferences, as potential confounders—such as age, medication use, and co-morbid conditions—could influence both dietary patterns and microbiome composition. In addition, no structured food frequency questionnaire or multi-day dietary recall was administered, resulting in a bias in the reported microbial ratios for fiber intake, macronutrient balance, or restrictive eating patterns that are common in ASD. This restricted our ability to disentangle microbiome signals that arise from neurodevelopmental status versus those driven by diet. Finally, while we observed shifts in key microbial ratios (e.g., F/B, A/P, and P/B), we did not integrate complementary approaches such as metabolomic or proteomic analyses. Such data could provide mechanistic insights into how these microbial changes translate into neurodevelopmental and behavioral outcomes.

## 5. Conclusions

Our study provides evidence that altered gut microbiota in children with ASD is associated with gastrointestinal dysfunction and may contribute to neurodevelopmental and behavioral impairments. We observed significant changes in microbial composition, including reduced F/B and A/P ratios, particularly in early childhood (0–4 years), suggesting a pro-inflammatory gut environment. Elevated levels of Proteobacteria further support this hypothesis, while the variability in the P/B ratio may reflect age-dependent or dietary factors. Although the cross-sectional design limits causal inference, our findings underscore the potential of microbiome-based interventions as adjunct therapies for ASD. To our knowledge, this is the first study to examine the role of these three key ratios as a potential biomarker, opening the possibility for novel diagnostic methods. Future longitudinal and interventional studies incorporating multi-omic approaches are needed to further elucidate the bidirectional gut–brain mechanisms in autism.

## Data Availability

The original contributions presented in this study are included in the article/Supplementary Materials. Further inquiries can be directed to the corresponding author.

## Supplementary Materials

The following supporting information can be downloaded at: https://www.mdpi.com/article/10.3390/nu17111775/s1, File S1: Statistical analysis performed for each of the three ratios.
