# Supplementary material for: Age-Dependent Gut Microbiome Dysbiosis in Autism Spectrum Disorder and the Role of Key Bacterial Ratios
Source: Nutrients. 2025 May 23;17(11):1775. doi: 10.3390/nu17111775 (PMC12157781; doi:10.3390/nu17111775)
Supplement: Supplementary file 1 [file nutrients-17-01775-s001.zip › nutrients-3633651-supplementary.pdf]

Supplementary file S1: statistical analysis performed for each of the three ratios.

Actinobacteria : Proteobacteria ratio

Age group	Mean ratio	SD ratio	Count	Meta mean	Cohen's d	% below meta	% above meta	% tied meta	*t*-test *p*	Wilcoxon *p*	Binomial *p*
0–4	0.71	1.1	26	11.92	-10.16	100	0	0	5.86E-27	2.98E-08	2.98E-08
4–6	0.67	1.61	81	2.61	-1.2	0.95	0.05	0	2.49E-17	1.9E-12	1.45E-18
6–8	0.69	1.41	84	2.7	-1.43	0.95	0.05	0	6.9E-22	8.07E-12	2.1E-19
8–10	0.78	1.79	64	2.23	-0.81	0.92	0.08	0	1.55E-08	1.78E-09	9E-13
10+	0.54	0.67	47	0.77	-0.35	0.7	0.3	0	0.02	0.0085	0.00794
European	0.68	1.44	302	2.69	-1.39	0.95	0.05	0	1.46E-72	3E-42	1.14E-67

Firmicutes : Bacteroidetes ratio

Age group	Mean ratio	SD ratio	Count	Meta mean	Cohen's d	% below meta	% above meta	% tied meta	*t*-test *p*	Wilcoxon *p*	Binomial *p*
0–4	1.18	1.52	26	5.67	-2.96	0.96	0.04	0	4.72E-14	5.96E-08	8.05E-07
4–6	1.22	1.58	80	1.54	-0.2	0.88	0.12	0	0.07	8.03E-09	3.16E-12
6–8	0.87	0.67	84	0.76	0.17	0.48	0.5	0.02	0.12	0.86	0.91
8–10	0.99	0.66	64	1.61	-0.94	0.84	0.16	0	2.11E-10	1.25E-07	2E-08
10+	0.99	0.82	47	0.87	0.14	0.6	0.4	0	0.33	0.43	0.24
European	1.03	1.09	301	3.21	-1.99	0.97	0.03	0	1.2E-106	4.45E-45	7.68E-76

Prevotella : Bacteroides ratio

Age group	Mean ratio	SD ratio	Count	Meta mean	Cohen's d	% below meta	% above meta	% tied meta	*t*-test *p*	Wilcoxon *p*	Binomial *p*
0–4	0.55	1.66	24	0.18	0.23	0.79	0.21	0	0.28	0.1	0.00661
4–6	0.44	1.59	66	0.7	-0.16	0.92	0.08	0	0.19	6.12E-08	2.63E-13
6–8	1.2	3.52	51	0.13	0.31	0.71	0.29	0	0.03	0.98	0.0046
8–10	0.7	1.53	33	0.67	0.02	0.76	0.24	0	0.91	0.1	0.00455
10+	2.44	3.98	20	0.54	0.48	0.5	0.5	0	0.05	0.25	1
European	0.91	2.59	194	0.54	0.14	0.79	0.21	0	0.05	7.54E-06	2.14E-16
